# The effect of joint angles on shot performance during the swing with different clubs among female professional golfers

**DOI:** 10.3389/fspor.2026.1867187

**Published:** 2026-06-22

**Authors:** Yuyang Zhang, Heng Liu, Chao Liu

**Affiliations:** 1 School of Leisure and Digital Sports, Guangzhou Sport University, Guangzhou, China; 2 School of Sports Training, Tianjin University of Sport, Tianjin, China

**Keywords:** different clubs, female, golf, joint angles, pro-golfer, shot performance

## Abstract

**Objective:**

This study aimed to investigate the differences in body joint angles during driver, 5-iron, and 7-iron swings in female professional golfers and their relationships with shot performance, providing a biomechanical basis for technical training.

**Methods:**

Twelve female professional golfers were recruited. A Qualisys three-dimensional motion capture system (250 Hz) and a FlightScope Xi launch monitor were used to collect data at four key swing phases: half backswing (HB), top of backswing (TOP), half downswing (HD), and impact (IMP). Joint angles of the head, neck, torso, pelvis, elbow, wrist, and knee were measured, along with performance parameters including carry, clubhead speed (CHS), smash factor, vertical angle, axis, and backspin rate (RPM). One-way repeated-measures ANOVA was used to compare differences among clubs, and Pearson correlation analysis was performed to examine the relationships between joint angles and shot performance.

**Results:**

The driver produced significantly greater carry, CHS, and smash factor than the irons (*p* < 0.001). The 7-iron showed significantly greater vertical angle and RPM than the driver and 5-iron (*p* < 0.05). During the driver, head forward tilt and rotation at HB and TOP were significantly smaller than irons (*p* < 0.05), while torso rotation at IMP was significantly greater (*p* < 0.05). The 5-iron exhibited the smallest lead knee internal rotation during the backswing and insufficient lead knee flexion at HD (*p* < 0.05). No significant differences were found in lead elbow and wrist (*p* > 0.05). Correlation analysis revealed that lead forearm pronation at TOP was negatively correlated with CHS during the driver swing (*r* = −0.789), lead knee external rotation at IMP was positively correlated with smash factor (*r* = 0.652), torso forward tilt at IMP was negatively correlated with carry distance during the 5-iron swing (*r* = −0.653), and lead knee flexion at HD was positively correlated with smash factor during 7-iron (*r* = 0.799).

**Conclusions:**

Female professional golfers adapt to club-specific hitting demands primarily through adjustments in head, torso, and knee joint angles, while maintaining a relatively stable upper limb control pattern across club types. From a practical standpoint, driver training should emphasize lead forearm supination and lead knee external rotation braking at IMP; 5-iron training should prioritize control of torso forward tilt and head side tilt; and 7-iron training should focus on maintaining lead knee flexion and promoting sequential separation between pelvis and torso rotation.

## 1 Introduction

The golf swing is a highly precise motor skill in which technical consistency and accuracy are fundamental determinants of shot performance (1, 2). Unlike many other sports, the golf swing is completed in approximately 1.5 s, yet demands precise multi-joint coordination throughout the entire body; even minor technical deviations can result in substantial errors in ball flight (3, 4). Previous research has established that the angular configurations of multiple body segments including the head, neck, torso, pelvis, elbow, wrist, and knee are associated with swing mechanics and ball shotting quality (5). As competitive female's professional golf continues to evolve, the demand for technical refinement among female professionals has grown considerably. Understanding the joint angle regulation patterns underlying the swing in this population therefore carries important practical implications for performance enhancement. Golfers adjust address posture, ball position, and trunk inclination according to club characteristics, but high skilled golfers tend to preserve relatively consistent core movement patterns after swing initiation (6). Different club types vary substantially in structural characteristics and functional demands, requiring golfers to adapt their body posture and precisely modulate joint angles to achieve distinct ball flight objectives (7). The driver, designed to maximize distance, features the longest shaft and largest swing arc, necessitating greater rotational amplitude to accumulate kinetic energy (8). The 7-iron, which prioritizes precision and green-stopping ability, has a larger loft angle and places higher demands on postural balance and joint control accuracy (9). The 5-iron, bridging distance and accuracy, imposes more comprehensive requirements on joint angle regulation (10). Despite these functional distinctions, quantitative research on club-specific swing kinematics in female professional golfers remains limited, constraining our understanding of their technical characteristics. Accordingly, this study aims to identify which joint angles change across clubs and which remain stable. However, these variable specific patterns in female professional golfers remain poorly understood.

The head and neck serve as the structural foundation for the integration of visual and vestibular inputs, where stability is paramount for maintaining a consistent gaze on the target to ensure ball shotting accuracy. Vickers quiet eye theory posits that the duration of visual fixation prior to movement execution is a decisive factor in motor performance; excessive head movement disrupts the vestibular system, leading to gaze deviation and clubface control errors (11). Studies have demonstrated that elite golfers exhibit significantly smaller changes in head forward tilt and rotation throughout the swing compared to amateur players, a stability that facilitates postural balance and precise spatial perception (12). Joyce et al. reported significant three-dimensional torso kinematic differences between the driver and 5-iron, with the driver eliciting substantially greater torso rotational amplitude (13). Further research has revealed that a larger differential between torso and pelvis rotation at the top of the backswing is a key determinant of driving distance in skilled golfers, and that peak torso rotational velocity occurs approximately 50–70 ms after peak pelvis rotational velocity which a temporal separation that provides the biomechanical basis for the “whip-effect” energy transfer observed in elite performers (14). The kinematics of the lead arm (left upper arm in right-handed golfers) directly govern clubface control and energy transfer efficiency (15). Given the research focus and practical orientation of this study, we prioritized discrete joint posture analysis rather than continuous time-series kinematic parameters. Precise regulation of lead forearm rotation at IMP is essential for achieving a square clubface at ball contact, as excessive forearm rotation compromises clubface stability. Wrist flexion-extension and radio ulnar deviation are regulated to maintain club lag, thereby prolonging the duration of force application (16). The knee joint plays a pivotal role in balance maintenance and power transmission throughout the swing. Stokes et al. reported that lead knee (left knee in right-handed golfers) flexion and rotation angles directly influence weight transfer timing and pelvis clearance, which in turn affect both carry and directional control (17). However, existing research has predominantly focused on male golfers; female golfers exhibit significantly lower angular velocities at the wrist and elbow joints compared to their male counterparts, suggesting the existence of sex-specific swing strategies (18), yet systematic kinematic quantification in this population remains lacking.

Shot performance is determined by the interplay of multiple biomechanical factors (18, 19). Carry is primarily governed by CHS and smash factor (the ratio of ball speed to CHS), both of which are influenced by club & ball impact conditions and broader swing mechanics (5, 20). Accordingly, the present study aimed to identify joint-angle patterns that may serve as accessible indicators for technique optimization in female professional golfers. Vertical angle and RPM are primarily determined by club& ball impact conditions, including dynamic loft, angle of attack, face orientation, and impact location. In this study, the selected joint-angle variables may influence shot performance indirectly by altering swing posture, segmental sequencing, and club delivery at impact. Accordingly, greater dynamic loft and favorable impact location may be associated with higher launch angle and RPM through the vertical gear effect and gyroscopic stability mechanism (21). Axis reflects the alignment between clubface orientation and swing path and serves as the key parameter for directional control (22). Existing research has provided preliminary evidence that joint angles are associated with shot performance: torso rotation amplitude at the top of the backswing is positively correlated with clubhead speed (13), and lead knee flexion during the downswing influences shotting consistency (23). Nevertheless, several important gaps remain. First, the majority of studies have focused on male professional or amateur golfers (7, 24–26), overlooking the distinct characteristics of female professionals with respect to muscular strength and joint range of motion; the joint angle regulation strategies of female golfers may differ fundamentally from those of their male counterparts. Second, existing investigations have largely examined a single club type or a single joint, without systematically exploring multi-joint angle differences across commonly used clubs at key swing moments or their associations with shot performance in female professionals. Third, the multi-joint coordinative mechanisms underlying the swing in female professional golfers remain insufficiently characterized, precluding the provision of precise, individualized training guidelines. Therefore, the present study recruited female professional golfers and employed a three-dimensional motion capture system to quantify joint angles of the head, neck, torso, pelvis, elbow, wrist, and knee at four key swing moments (HB, TOP, HD and IMP) during driver, 5-iron, and 7-iron. The associations between these joint angles and shot performance, including carry, CHS, smash factor, vertical angle, axis, and RPM, were subsequently examined. The findings are intended to provide biomechanical evidence to support club-specific technical training in female professional golfers.

## 2 Materials and methods

### 2.1 Subjects

Twelve right-handed female professional golfers (age: 24.58 ± 4.11 years; golf experience: 7.42 ± 2.36 years; height: 1.70 ± 0.04 m; body mass: 62.00 ± 9.16 kg) were recruited to participate in this study. Inclusion criteria were: (1) no major musculoskeletal injury in the preceding 6 months and no chronic pain affecting swing mechanics; (2) no vigorous physical activity within 24 h prior to testing; and (3) technically stable swing mechanics and good physical condition on the day of testing. Exclusion criteria were: (1) acute muscle or ligament injury within the 3 months preceding the study; (2) current pregnancy or within 6 months postpartum; (3) recent use of performance-altering medications or supplements; (4) consumption of analgesic medications or alcohol within 24 h prior to testing; and (5) inability to complete the full testing protocol. The study was approved by the Ethics Committee of Tianjin University of Sport (approval number: TJUS2025-111), and all participants provided written informed consent prior to data collection. Participant recruitment was conducted from October 20 to November 30, 2025.

### 2.2 Testing protocol

**Figure 1:**
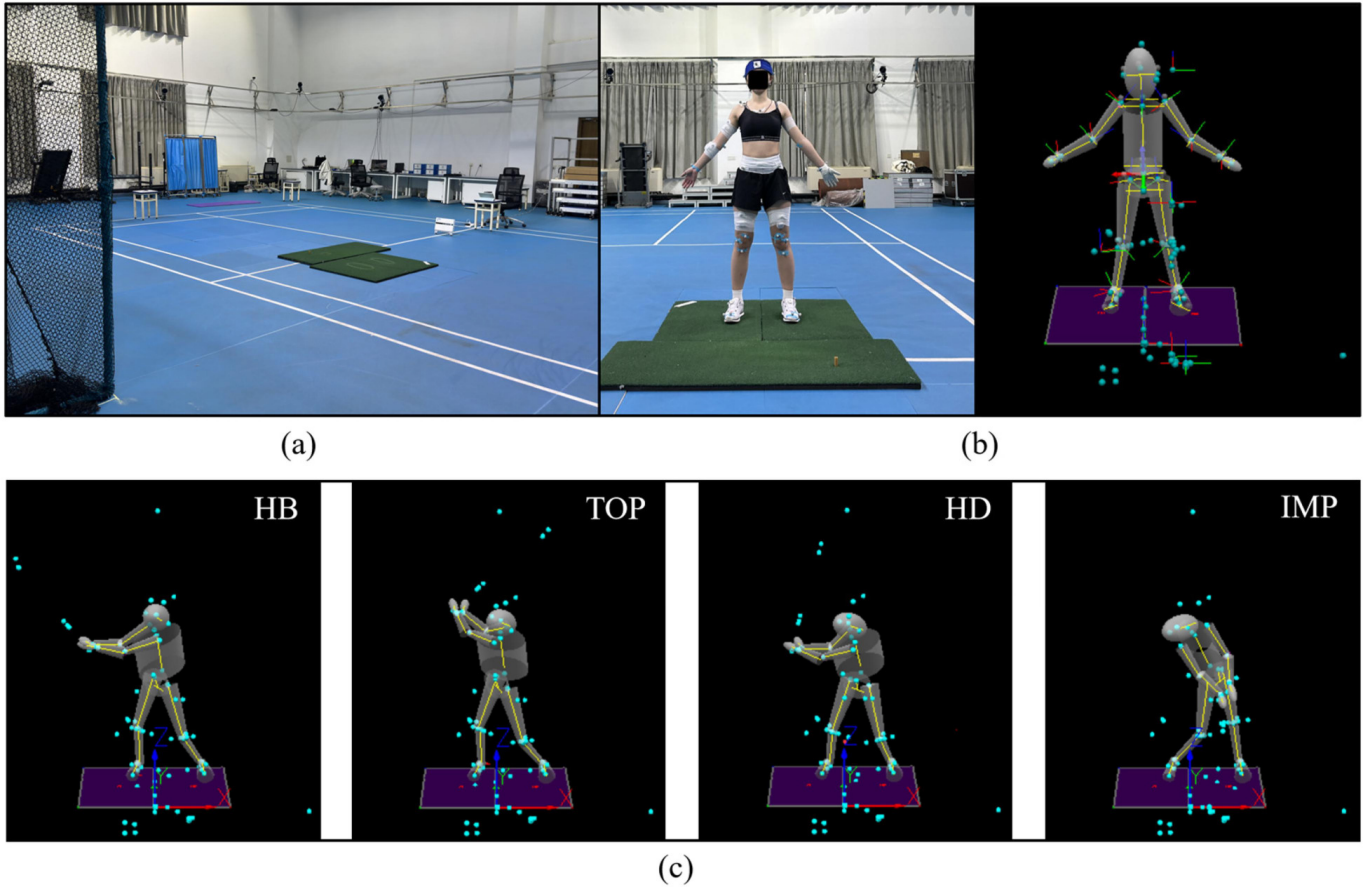
Experimental setup and swing moment. **(a)** Experimental setup, **(b)** mark placement and local segment coordinate system, **(c)** the moments of swing. HB refers to the lead arm moves clockwise to the horizontal plane; TOP refers to the angular velocity of the club is 0; HD refers to the lead arm moves counterclockwise to the horizontal plane; IMP refers to one frame before the club head hits the ball.

A cross-sectional design was employed. The independent variable was club type (driver, 5-iron, and 7-iron). Dependent variables comprised joint angles of the head, neck, torso, pelvis, elbow, wrist, and knee at four key swing moments as well as shot performance including carry, CHS, smash factor, launch angle, axis, and RPM. All participants used their own competition-standard clubs and equipment. Testing was conducted in a laboratory setting within an indoor hitting cage measuring 3.15 m (height) × 1.6 m (width) × 2.8 m (length). A golf hitting mat was positioned at least 4.5 m from the cage netting. A FlightScope Xi launch monitor (FlightScope, USA) was placed directly behind the ball position at a distance of no less than 3 m to record ball flight data. The launch monitor was aligned with the target line according to the manufacturer's indoor setup protocol and calibrated before data collection to ensure correct ball-flight tracking under indoor conditions. The experimental setup is illustrated in Figure 1a. Prior to data collection, participants completed a self-directed warm-up of approximately 10 min to acclimate to the laboratory environment. Retroreflective markers were subsequently attached to anatomical landmarks according to the Visual3D Golf model, including the head, neck, trunk, pelvis, upper limbs, forearms, hands, thighs, shanks, and feet (Figure 1b). Three-dimensional kinematic data were captured using a Qualisys motion capture system (Qualisys Track Manager, Sweden) at a sampling frequency of 250 Hz. The motion-capture system and launch monitor were temporally synchronized using a common time reference, and the impact instant was aligned across systems for subsequent analyses. Each participant performed full swings with the driver, 5-iron, and 7-iron on the hitting mat. A minimum of three successful trials were collected per club, with a 30-s rest interval between consecutive swings and a 5-min rest interval between club conditions. Club testing order was randomized to minimize learning effects. A trial was considered successful if all of the following criteria were met: (1) complete marker trajectory data with no missing markers throughout the swing; (2) carry distance within ±10% of the participant's mean carry distance (Measured prior to the formal experiment, driver: 186.02 ± 11.55 yards; 5-iron: 141.97 ± 12.85 yards; 7-iron: 130.26 ± 9.77 yards) for that club; (3) directional deviation of less than 5° from the target line; and (4) complete and valid data recorded by the launch monitor.

### 2.3 Data processing

Raw marker trajectory data were preprocessed using QTM software. Marker trajectories were first inspected for labeling errors and artifacts. Short-duration gaps (<10 frames) in marker trajectories were filled using cubic spline interpolation. Processed data were exported in C3D format. Biomechanical variables were subsequently computed using the Golf model in Visual3D software (C-Motion Inc., USA). Segment coordinate systems were defined according to the Visual3D Golf model, and joint angles were calculated using a consistent Cardan rotation sequence for each segment, with forward tilt, side tilt, and rotation defined relative to the laboratory coordinate system. Kinematic data were filtered in Visual3D (C-Motion) using a Butterworth low-pass filter. The cutoff frequencies were set at 10 Hz for the backswing and 20 Hz for the downswing according to the Qualisys default settings for the golf-specific model (5). Four discrete swing events were identified for joint angle analysis (Figure 1c). Ball flight parameters were exported directly in CSV format via the FS Golf application (FlightScope, USA).

### 2.4 Statistical analysis

Data aggregation and management were performed using Microsoft Excel 2021 (Microsoft, USA). The mean value of each participant's valid trials was used as the representative score for statistical analysis. All statistical procedures were conducted using SPSS version 27.0 (IBM, USA). The Shapiro–Wilk test was applied to assess the normality of all variables; all data were confirmed to be normally distributed. One-way repeated-measures ANOVA was used to compare ball flight parameters and joint angles across the three club conditions. Effect sizes were calculated using partial eta-squared $\left(\eta_{p}^{2}\right)$. Mauchly's test of sphericity was applied to assess the analysis. When the sphericity assumption was violated, Greenhouse-Geisser correction was applied to adjust the degrees of freedom. Bonferroni-adjusted pairwise comparisons were used for *post hoc* analyses. Pearson correlation coefficients were calculated to examine the relationships between joint angles and ball flight parameters. Statistical significance was set at *α* = 0.05.

## 3 Results

### 3.1 Shot performance

**Figure 2:**
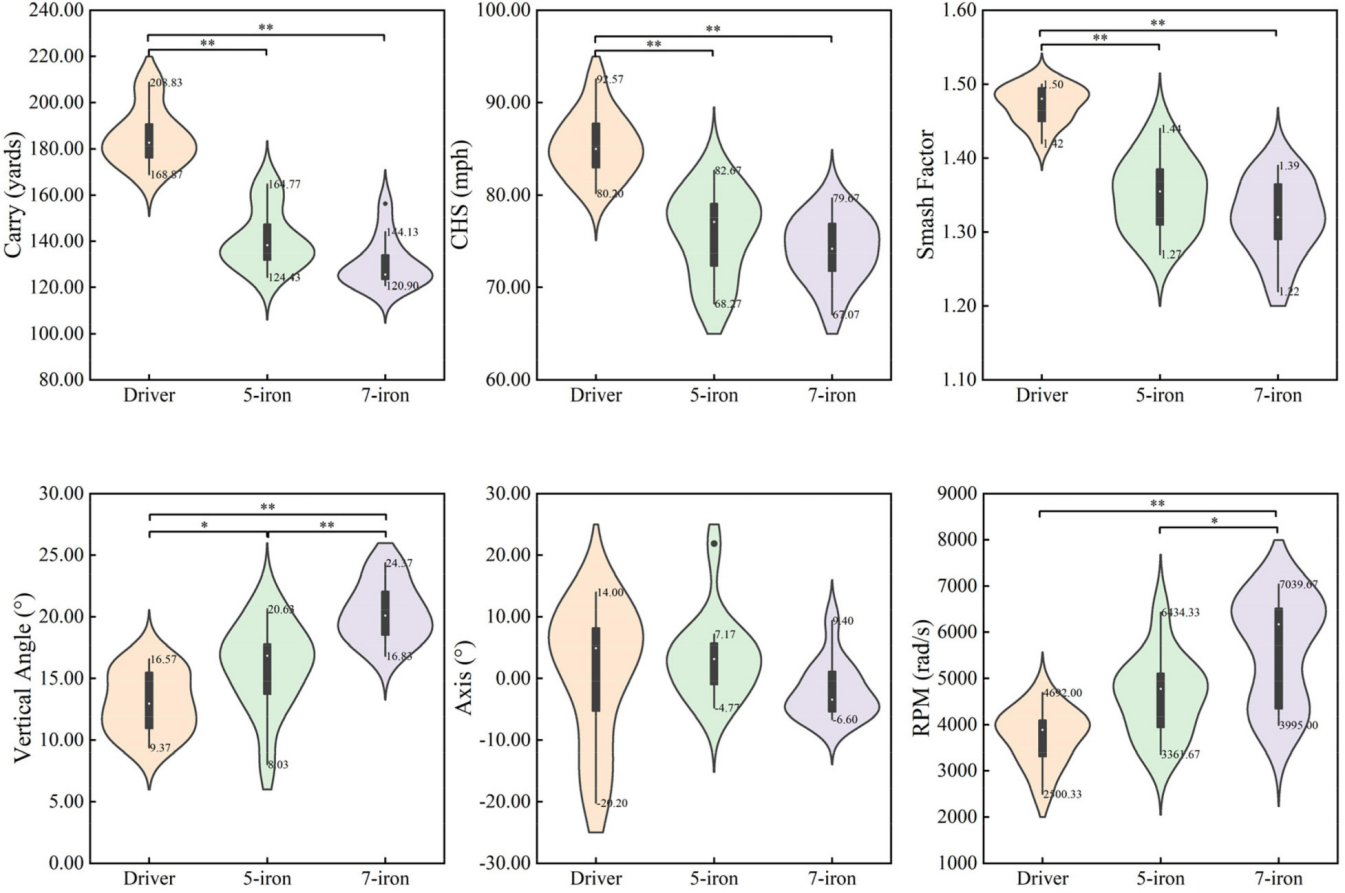
Comparison of performance parameters for different clubs. Carry refers to the distance the ball travels through the air until it first lands; CHS refers to the instantaneous linear velocity of the clubhead at impact; smash factor refers to the ratio of ball speed to CHS, reflecting the club's energy transfer efficiency; vertical Angle refers to the angle between the ball's flight path and the radar's horizontal plane; axis refers to the angle of the ball's spin axis, reflecting the degree of side spin (positive for right spin); RPM refers to the rate of rotation around a fixed axis.

Depending on the club's design and intended use, there can be significant differences in performance parameters. In the one-way ANOVA analysis, there was a significant difference on carry (*p* < 0.001, $\eta_{p}^{2}=0.801$). A Tukey HSD *post-hoc* analysis showed that carry for the driver was significantly greater than 5-iron (185.27 ± 12.41 vs. 140.88 ± 12.74, *p* < 0.001) and greater than 7-iron (185.27 ± 12.41 vs. 130.37 ± 10.54, *p* < 0.001). There was a significant difference on CHS (*p* < 0.001, $\eta_{p}^{2}=0.611$). The CHS of the driver was significantly greater than 5-iron (85.39 ± 3.46 vs. 75.89 ± 4.66, *p* < 0.001) and greater than 7-iron (85.39 ± 3.46 vs. 74.12 ± 3.56, *p* < 0.001). There was a significant difference on smash factor (*p* < 0.001, $\eta_{p}^{2}=0.696$). The smash factor of the driver was significantly greater than 5-iron (1.47 ± 0.03 vs. 1.35 ± 0.05, *p* < 0.001) and greater than 7-iron (1.47 ± 0.03 vs. 1.32 ± 0.05, *p* < 0.001). There was a significant difference on vertical angle (*p* < 0.001, $\eta_{p}^{2}=0.522$). The vertical angle of the driver was significantly smaller than 5-iron (13.04 ± 2.51 vs. 16.01 ± 3.51, *p* = 0.040) and smaller than 7-iron (13.04 ± 2.51 vs. 20.38 ± 2.41, *p* < 0.001); the 5-iron smaller than 7-iron (16.01 ± 3.51 vs. 20.38 ± 2.41, *p* = 0.002). As shown in Figure 2, there was no difference on axis (*p* > 0.05). There was a significant difference on RPM (*p* < 0.001, $\eta_{p}^{2}=0.413$). The RPM of the 7-iron was significantly greater than driver (5,633.25 ± 1,136.84 vs. 3,747.40 ± 608.69, *p* < 0.001) and greater than 5-iron (5,633.25 ± 1,136.84 vs. 4,621.56 ± 863.50, *p* = 0.024).

### 3.2 Head and neck

**Figure 3:**
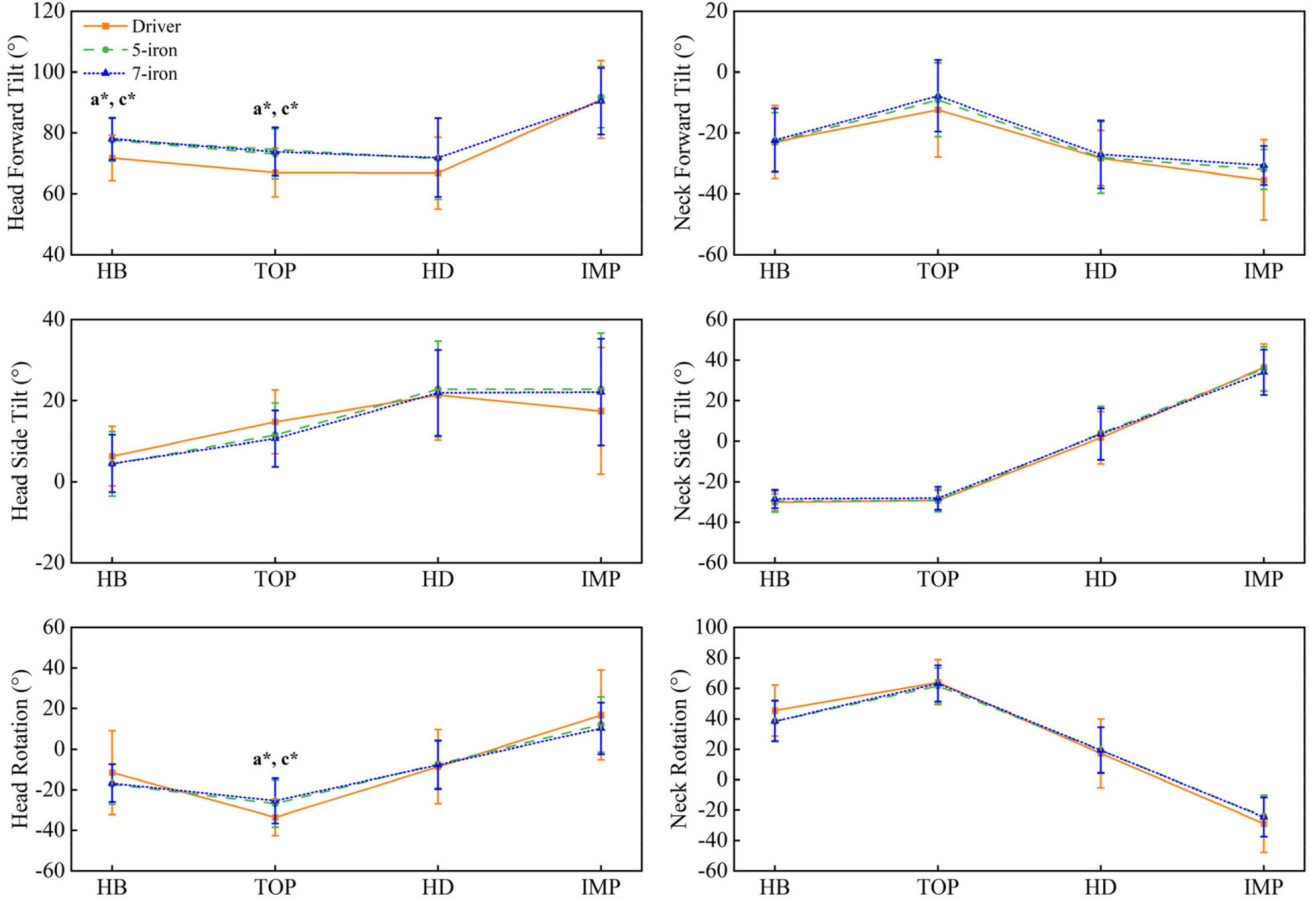
Head and neck joint angles at various swing moments for different clubs. Forward Tilt refers to the *X*-component of rotation of a body segment relative to the virtual laboratory, forward tilt is positive; side Tilt refers to the *Y*-component of rotation of a body segment relative to the virtual laboratory, tilt away from the target direction is positive; rotation refers to the *Z*-component of rotation of a body segment relative to the virtual laboratory, rotation toward the target direction is positive. a* Significant differences between driver and 5-iron; c* Significant differences between driver and 7-iron.

As shown in Figure 3, at HB, a significant difference was observed in Head Forward Tilt (*p* = 0.036, $\eta_{p}^{2}=0.171$). Head Forward Tilt was significantly smaller in the driver than 5-iron (71.79 ± 7.44 vs. 77.72 ± 7.05, *p* = 0.032) and 7-iron (71.79 ± 7.44 vs. 78.10 ± 6.92, *p* = 0.019). At this moment, no significant difference was found in Head Rotation among clubs (*p* = 0.568), whereas the mean value of Head Rotation was greater in the driver than 5-iron (5.63°) and 7-iron (5.16°). At TOP, a significant difference was detected in Head Forward Tilt (*p* = 0.028, $\eta_{p}^{2}=0.237$). Head Forward Tilt was significantly smaller in the driver than 5-iron (67.02 ± 8.06 vs. 73.17 ± 8.19, *p* = 0.026) and 7-iron (67.02 ± 8.06 vs. 73.88 ± 7.93, *p* = 0.031). Meanwhile, Head Rotation was significantly difference (*p* = 0.039, $\eta_{p}^{2}=0.163$). Head Rotation was significantly smaller in the driver than 5-iron (−33.59 ± 9.05 vs. −26.77 ± 11.59, *p* = 0.048) and 7-iron (−33.59 ± 9.05 vs. −25.39 ± 11.10, *p* = 0.035). At HD, no significant difference was found in Head Forward Tilt (*p* = 0.558), but the mean value of Head Forward Tilt in the driver was smaller than 5-iron (4.71°) and 7-iron (5.07°). At IMP, no significant difference was observed in Head Side Tilt (*p* = 0.611), while the mean value of Head Side Tilt in the driver was smaller than 5-iron (5.34°) and 7-iron (4.64°).

### 3.3 Torso and pelvis

**Figure 4:**
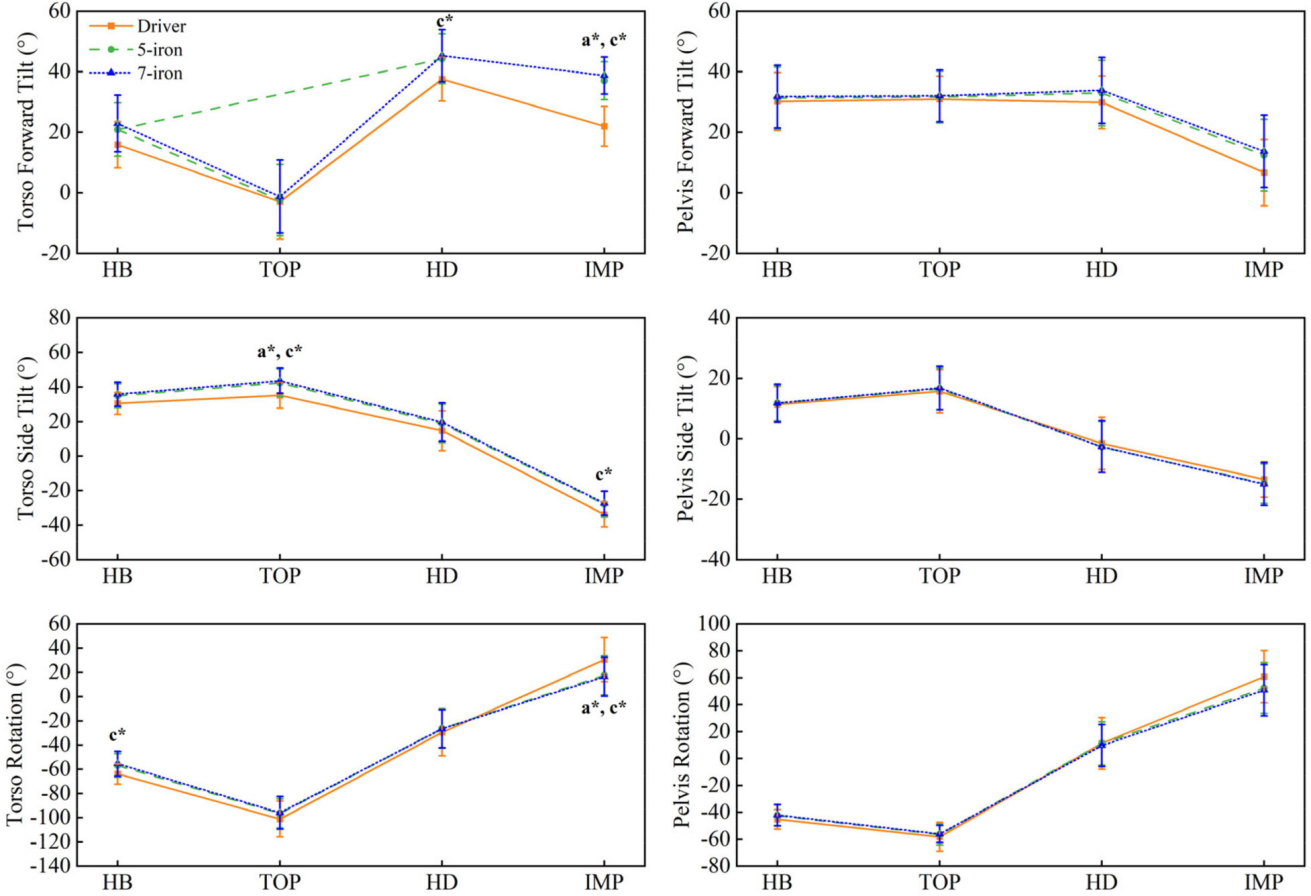
Torso and pelvis joint angles at various swing moments for different clubs. Forward Tilt refers to the *X*-component of rotation of a body segment relative to the virtual laboratory, forward tilt is positive; side Tilt refers to the *Y*-component of rotation of a body segment relative to the virtual laboratory, tilt away from the target direction is positive; rotation refers to the *Z*-component of rotation of a body segment relative to the virtual laboratory, rotation toward the target direction is positive. a* Significant differences between driver and 5-iron; c* Significant differences between driver and 7-iron.

As shown in Figure 4, at HB, a significant difference was observed in Torso Rotation (*p* = 0.041, $\eta_{p}^{2}=0.182$). Torso Rotation was significantly smaller in the driver than in the 7-iron (−63.82 ± 8.38 vs. −55.40 ± 10.17, *p* = 0.029). At this moment, no significant difference was found in Torso Forward Tilt (*p* = 0.141), whereas the mean value of Torso Forward Tilt was smaller in the driver than 5-iron (5.00°) and 7-iron (6.98°). At TOP, a significant difference was detected in Torso Side Tilt (*p* = 0.020, $\eta_{p}^{2}=0.212$). Torso Side Tilt was significantly smaller in the driver than 5-iron (35.16 ± 7.36 vs. 42.39 ± 7.92, *p* = 0.042) and 7-iron (35.16 ± 7.36 vs. 43.62 ± 7.26, *p* = 0.025). Meanwhile, no significant difference was found in Torso Rotation (*p* = 0.595), but the mean value of Torso Rotation in the driver was smaller than 5-iron (4.61°) and 7-iron (5.16°). At HD, a significant difference was observed in Torso Forward Tilt (*p* = 0.048, $\eta_{p}^{2}=0.168$). Torso Forward Tilt was significantly smaller in the driver than 7-iron (37.53 ± 7.23 vs. 45.28 ± 8.57, *p* = 0.050). At IMP, significant differences were found in Torso Forward Tilt, Torso Side Tilt, and Torso Rotation. For Torso Forward Tilt (*p* < 0.001, $\eta_{p}^{2}=0.605$), which were significantly smaller in the driver than 5-iron (22.02 ± 6.57 vs. 37.03 ± 6.29, *p* < 0.001) and 7-iron (22.02 ± 6.57 vs. 38.74 ± 6.14, *p* < 0.001). For Torso Side Tilt (*p* = 0.033, $\eta_{p}^{2}=0.184$), the driver showed significantly smaller than 7-iron (−33.75 ± 7.21 vs. −27.32 ± 6.91, *p* = 0.044). For Torso Rotation (*p* = 0.029, $\eta_{p}^{2}=0.206$), the driver presented significantly greater than 5-iron (30.56 ± 18.10 vs. 17.58 ± 16.28, *p* = 0.015) and 7-iron (30.56 ± 18.10 vs. 16.43 ± 16.07, *p* = 0.018). At this moment, no significant difference was detected in Pelvis Rotation (*p* = 0.402), whereas the mean value of Pelvis Rotation was greater in the driver than 5-iron (8.36°) and 7-iron (9.92°). No significant differences were observed in joint angles at other moments; for details see Figure 4.

### 3.4 Lead elbow and wrist

This study found no significant differences in lead elbow and wrist angles (*p* > 0.05, Table 1). At HB, there was no significant difference in Lead Wrist Flexion (*p* = 0.750), whereas the mean value of Lead Wrist Flexion was smaller in the driver than 5-iron (3.27°) and 7-iron (3.21°). At TOP, no significant difference was detected in Lead Wrist Flexion (*p* = 0.590), but the mean value of Lead Wrist Flexion in the driver was smaller than 5-iron (4.85°) and 7-iron (2.20°). At HD, no significant difference was observed in Lead Wrist Flexion (*p* = 0.392), while the mean value of Lead Wrist Flexion in the driver was smaller than 5-iron (6.23°) and 7-iron (5.24°). No significant difference was found in Lead Wrist Radial Deviation (*p* = 0.926), yet the mean value of Lead Wrist Radial Deviation was smaller in the 5-iron than driver (1.55°) and 7-iron (3.29°).

**Table 1:** Lead elbow and wrist joint angles at various swing moments for different clubs.

Moment	Joint angle	Driver (°)	5-iron (°)	7-iron (°)	*F*	*p*	$\eta_{p}^{2}$
HB	Lead elbow flexion	28.04 ± 10.69	27.62 ± 11.14	28.69 ± 10.77	0.030	0.971	0.002
	Lead wrist flexion	−6.16 ± 10.51	−2.89 ± 13.70	−2.95 ± 11.58	0.290	0.750	0.017
	Lead wrist radial deviation	18.04 ± 16.33	20.37 ± 16.31	22.66 ± 15.28	0.250	0.780	0.015
	Lead forearm rotation	−0.96 ± 4.81	−1.28 ± 5.49	−1.86 ± 6.06	0.083	0.920	0.005
TOP	Lead elbow flexion	42.46 ± 9.51	41.34 ± 10.80	41.43 ± 10.19	0.045	0.956	0.003
	Lead wrist flexion	−1.17 ± 10.19	3.68 ± 14.61	1.03 ± 8.91	0.536	0.590	0.031
	Lead wrist radial deviation	27.66 ± 24.37	27.49 ± 20.58	32.04 ± 21.21	0.163	0.850	0.010
	Lead forearm rotation	0.00 ± 4.00	−2.40 ± 6.20	−2.25 ± 5.02	0.819	0.450	0.047
HD	Lead elbow flexion	41.58 ± 9.53	44.18 ± 9.22	44.14 ± 9.66	0.297	0.745	0.018
	Lead wrist flexion	−2.91 ± 13.03	3.32 ± 12.38	2.33 ± 9.73	0.964	0.392	0.055
	Lead wrist radial deviation	16.33 ± 22.26	14.78 ± 19.06	18.07 ± 20.30	0.077	0.926	0.005
	Lead forearm rotation	−3.63 ± 10.58	−1.16 ± 4.75	−2.43 ± 3.50	0.374	0.691	0.022
IMP	Lead elbow flexion	37.90 ± 10.69	35.30 ± 11.67	35.55 ± 10.53	0.205	0.816	0.012
	Lead wrist flexion	−20.21 ± 7.71	−20.89 ± 8.08	−21.93 ± 7.91	0.145	0.866	0.009
	Lead wrist radial deviation	13.13 ± 17.60	13.36 ± 16.72	13.79 ± 16.23	0.005	0.995	0.000
	Lead forearm rotation	1.15 ± 4.55	1.37 ± 4.11	1.20 ± 4.24	0.008	0.992	0.000

Elbow flexion refers to the rotation of the forearm relative to the upper arm about the frontal axis, flexion is positive; wrist flexion refers to the rotation of the hand relative to the forearm about the frontal axis in the sagittal plane, dorsiflexion is positive; wrist radial deviation refers to the rotation of the hand relative to the forearm about the ulnar axis in the frontal plane, radial deviation is positive; forearm rotation refers to the rotation of the radius about the ulna, the *Z*-component of rotation in the local wrist coordinate system; pronation is positive.

### 3.5 Lead knee and trail knee

At HB, a significant difference was found in Lead Knee Rotation (*p* = 0.036, $\eta_{p}^{2}=0.189$). Lead Knee Rotation was significantly smaller in the 5-iron than driver (3.11 ± 5.57 vs. 8.31 ± 7.35, *p* = 0.039) and 7-iron (3.11 ± 5.57 vs. 6.84 ± 8.69, *p* = 0.046). At TOP, a significant difference was detected in Lead Knee Rotation (*p* = 0.019, $\eta_{p}^{2}=0.237$). Lead Knee Rotation was significantly smaller in the 5-iron than driver (2.31 ± 7.21 vs. 6.98 ± 7.53, *p* = 0.023) and 7-iron (2.31 ± 7.21 vs. 6.01 ± 9.07, *p* = 0.036). At HD, a significant difference was observed in Lead Knee Flexion (*p* = 0.045, $\eta_{p}^{2}=0.163$). Lead Knee Flexion was significantly smaller in the 5-iron than driver (43.57 ± 12.16 vs. 49.67 ± 10.69, *p* = 0.042). At IMP, a significant difference was found in Lead Knee Rotation (*p* = 0.046, $\eta_{p}^{2}=0.163$). Lead Knee Rotation was significantly greater in the 5-iron than driver (3.08 ± 4.41 vs. −1.83 ± 4.30, *p* = 0.045). No significant differences were observed in joint angles at other moments; for details see Table 2.

**Table 2:** Lead knee and trail knee joint angles at various swing moments for different clubs.

Moment	Joint angle	Driver (°)	5-iron (°)	7-iron (°)	*F*	*p*	$\eta_{p}^{2}$
HB	Lead knee flexion	32.34 ± 9.87	32.07 ± 13.66	34.79 ± 10.91	0.201	0.819	0.012
	Lead knee rotation	8.31 ± 7.35^a^	3.11 ± 5.57^b^	6.84 ± 8.69	4.296	0.036	0.189
	Trail knee flexion	23.95 ± 6.78	22.06 ± 6.71	21.79 ± 7.29	0.346	0.710	0.021
	Trail knee rotation	0.02 ± 3.40	0.29 ± 3.47	0.36 ± 3.28	0.034	0.966	0.002
TOP	Lead knee flexion	46.07 ± 10.94	43.35 ± 15.46	46.75 ± 11.24	0.241	0.787	0.014
	Lead knee rotation	6.98 ± 7.53^a^	2.31 ± 7.21^b^	6.01 ± 9.07	4.503	0.019	0.237
	Trail knee flexion	31.89 ± 10.00	28.66 ± 9.72	28.48 ± 9.34	0.471	0.628	0.028
	Trail knee rotation	−1.96 ± 5.45	−1.04 ± 4.63	−1.19 ± 3.88	0.134	0.875	0.008
HD	Lead knee flexion	49.67 ± 10.69^a^	43.57 ± 12.16	46.32 ± 9.80	3.818	0.045	0.163
	Lead knee rotation	−2.67 ± 5.74	−6.63 ± 15.70	−1.98 ± 5.91	0.723	0.493	0.042
	Trail knee flexion	54.20 ± 10.96	51.70 ± 11.53	51.32 ± 11.63	0.227	0.798	0.014
	Trail knee rotation	3.55 ± 6.65	4.13 ± 7.01	4.14 ± 6.94	0.029	0.971	0.002
IMP	Lead knee flexion	16.92 ± 13.48	14.69 ± 12.47	15.93 ± 11.41	0.096	0.909	0.006
	Lead knee rotation	−1.83 ± 4.30^a^	3.08 ± 4.41	−1.50 ± 4.60	3.792	0.046	0.163
	Trail knee flexion	38.86 ± 11.18	37.97 ± 12.47	37.14 ± 12.99	0.059	0.943	0.004
	Trail knee rotation	7.70 ± 4.85	7.62 ± 5.66	7.51 ± 5.92	0.004	0.996	0.000

Knee flexion refers to the rotation of the lower leg around the coronal axis relative to the thigh, flexion is positive; knee rotation refers to the rotation of the tibia relative to the femur, it is the *Z*-component of rotation in the local knee joint coordinate system, and internal rotation is positive.

a Significant differences between driver and 5-iron.

b Significant differences between 5-iron and 7-iron.

### 3.6 Correlation analysis

#### 3.6.1 Correlation analysis of the driver

**Figure 5:**
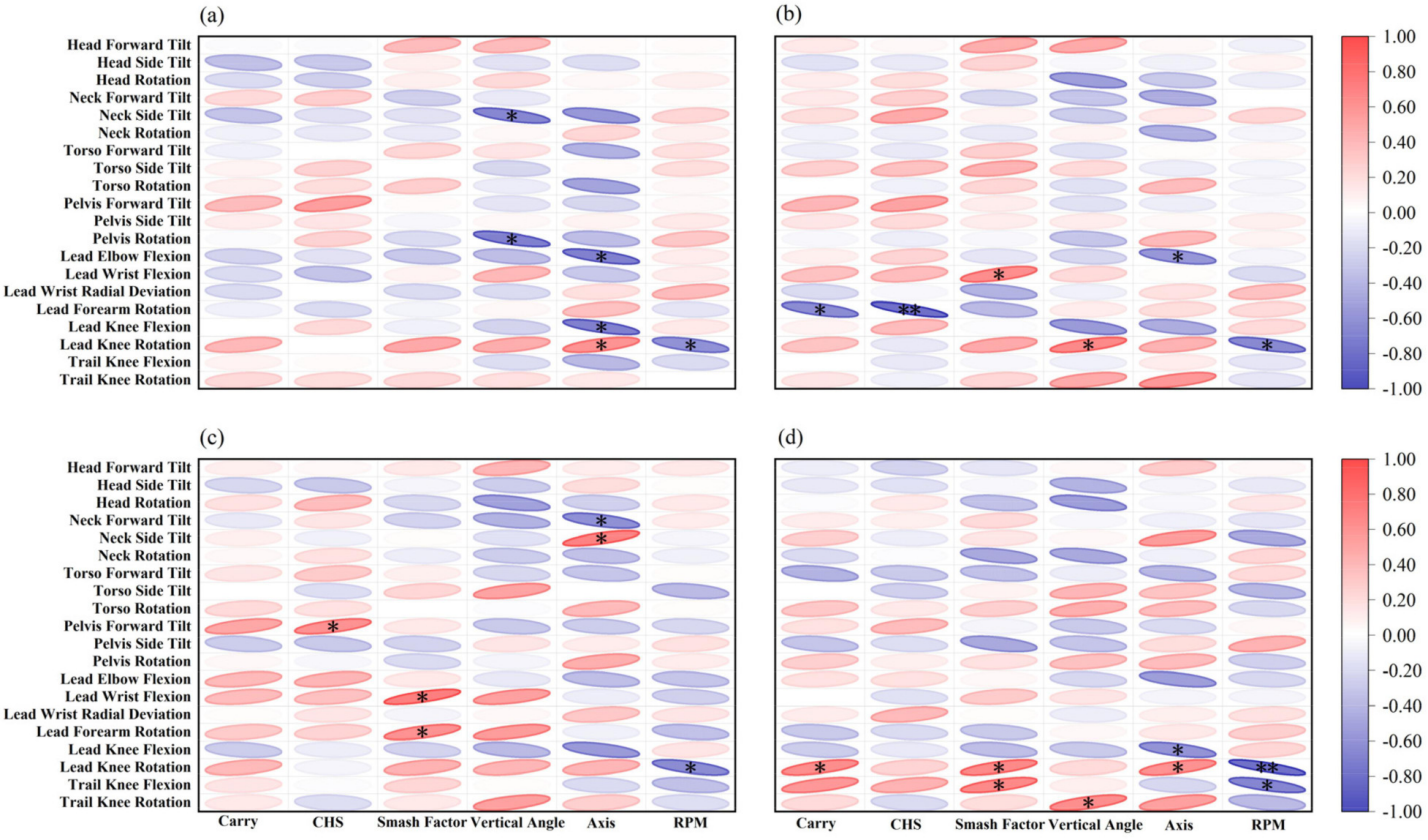
Correlation between joint angles and shot performance during driver swing. **(a)** Driver at HB, **(b)** Driver at TOP, **(c)** Driver at HD, **(d)** Driver at IMP. *Indicates *p* < 0.05, **Indicates *p* < 0.01; the same applies below.

For the driver, carry was negatively correlated with TOP-Lead Forearm Rotation (*r* = −0.634, *p* = 0.027) and positively correlated with IMP-Lead Knee Rotation (*r* = 0.623, *p* = 0.030). CHS was negatively correlated with TOP-Lead Forearm Rotation (*r* = −0.789, *p* = 0.002) and positively correlated with HD-Pelvis Forward Tilt (*r* = 0.611, *p* = 0.035). Smash factor was positively correlated with TOP-Lead Wrist Flexion (*r* = 0.612), HD-Lead Wrist Flexion (*r* = 0.702), HD-Lead Forearm Rotation (*r* = 0.610), IMP-Lead Knee Rotation (*r* = 0.652), and IMP-Trail Knee Flexion (*r* = 0.670) (all *p* < 0.05). Vertical Angle was negatively correlated with HB-Neck Side Tilt (*r* = −0.659) and HB-Pelvis Rotation (*r* = −0.686) (both *p* = 0.014), and positively correlated with TOP-Lead Knee Rotation (*r* = 0.650) and IMP-Trail Knee Rotation (*r* = 0.626) (both *p* < 0.05). Axis was negatively correlated with HB-Lead Elbow Flexion (*r* = −0.685), HB-Lead Knee Flexion (*r* = −0.697), TOP-Lead Elbow Flexion (*r* = −0.577), HD-Neck Forward Tilt (*r* = −0.600), and IMP-Lead Knee Flexion (*r* = −0.581) (all *p* < 0.05); and positively correlated with HB-Lead Knee Rotation (*r* = 0.584), HD-Neck Side Tilt (*r* = 0.686), and IMP-Lead Knee Rotation (*r* = 0.609) (all *p* < 0.05). RMP was negatively correlated with HB-Lead Knee Rotation (*r* = −0.592), TOP-Lead Knee Rotation (*r* = −0.652), HD-Lead Knee Rotation (*r* = −0.635), IMP-Lead Knee Rotation (*r* = −0.791), and IMP-Trail Knee Flexion (*r* = −0.666) (all *p* < 0.05). No significant correlations were found for joint angles at other moments; see Figure 5 for details.

#### 3.6.2 Correlation analysis of the 5-iron

**Figure 6:**
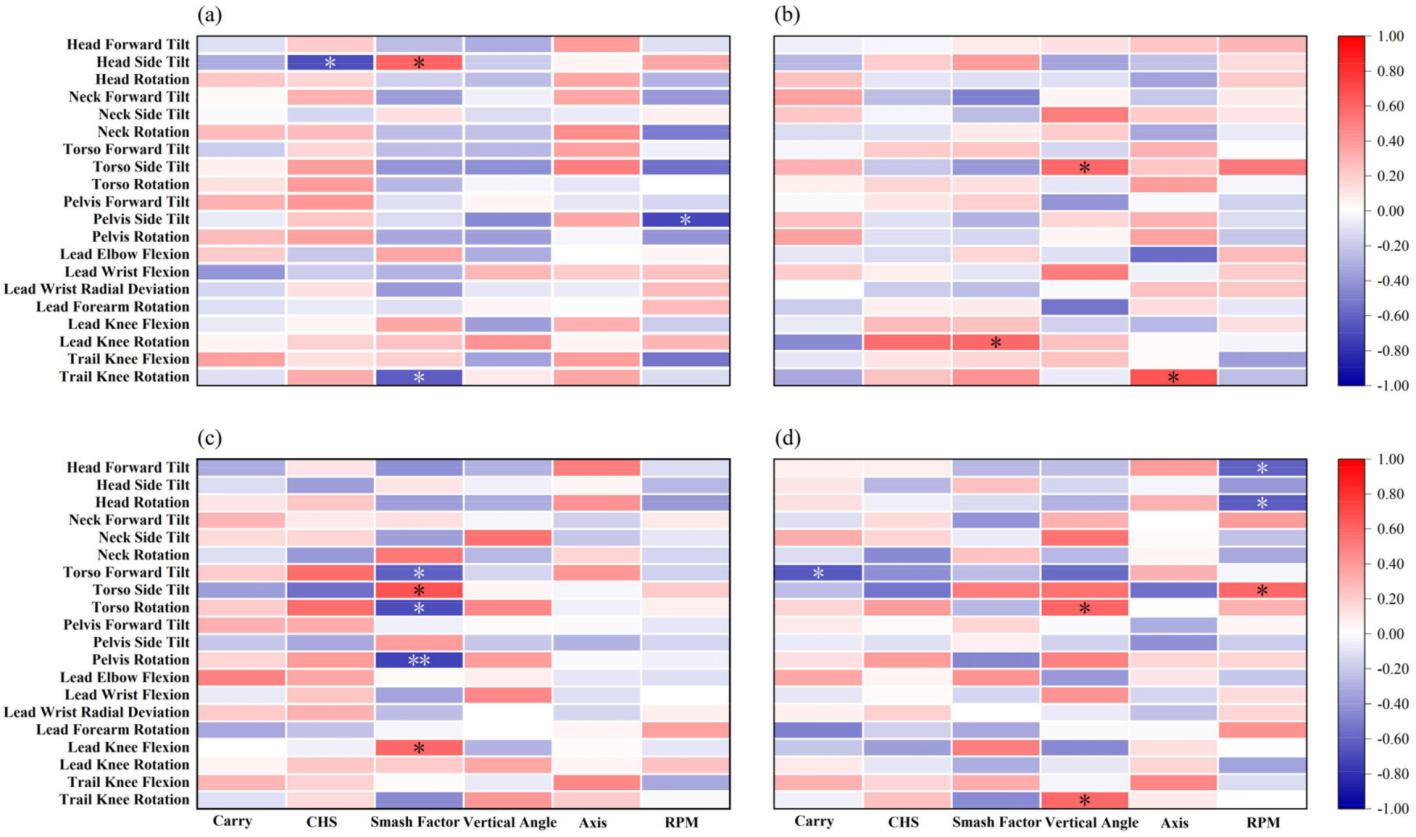
Correlation between joint angles and shot performance during 5-iron swing. **(a)** 5-iron at HB, **(b)** 5-iron at TOP, **(c)** 5-iron at HD, **(d)** 5-iron at IMP.

For the 5-iron, carry was negatively correlated with IMP-Torso Forward Tilt (*r* = −0.653, *p* = 0.021). CHS was negatively correlated with HB-Head Side Tilt (*r* = −0.695, *p* = 0.012). Smash factor was positively correlated with HB-Head Side Tilt (*r* = 0.618), TOP-Lead Knee Rotation (*r* = 0.624), HD-Torso Side Tilt (*r* = 0.645), and HD-Lead Knee Flexion (*r* = 0.586) (all *p* < 0.05); and negatively correlated with HB-Trail Knee Flexion (*r* = −0.619), HD-Torso Forward Tilt (*r* = −0.591), HD-Torso Rotation (*r* = −0.690), and HD-Pelvis Rotation (*r* = −0.729) (all *p* < 0.05). Vertical Angle was positively correlated with TOP-Torso Side Tilt (*r* = 0.703), IMP-Torso Rotation (*r* = 0.604), and IMP-Trail Knee Rotation (*r* = 0.580) (all *p* < 0.05). Axis was positively correlated with TOP-Trail Knee Rotation (*r* = 0.752, *p* = 0.014). RMP was negatively correlated with HB-Pelvis Side Tilt (*r* = −0.705), IMP-Head Forward Tilt (*r* = −0.619), and IMP-Head Rotation (*r* = −0.625) (all *p* < 0.05), and positively correlated with IMP-Torso Side Tilt (*r* = 0.589, *p* = 0.044). No significant correlations were found for joint angles at other moments; see Figure 6 for details.

#### 3.6.3 Correlation analysis of the 7-iron

**Figure 7:**
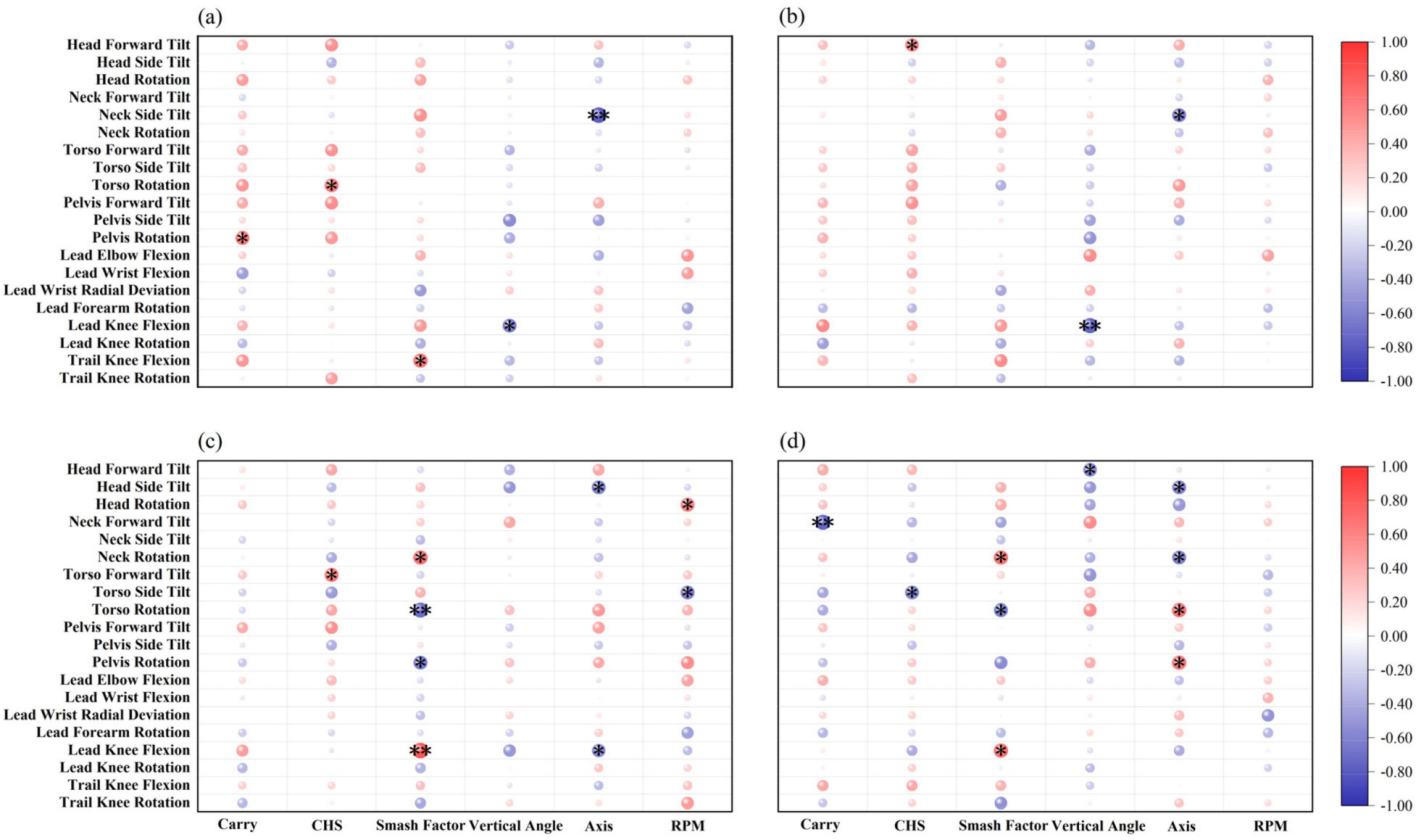
Correlation between joint angles and shot performance during 7-iron swing. **(a)** 7-iron at HB, **(b)** 7-iron at TOP, **(c)** 7-iron at HD, **(d)** 7-iron at IMP.

As shown in Figure 7, for the 7-iron, carry was positively correlated with HB-Pelvis Rotation (*r* = 0.621, *p* = 0.031) and negatively correlated with IMP-Head Forward Tilt (*r* = −0.743, *p* = 0.006). CHS was positively correlated with HB-Torso Rotation (*r* = 0.621), TOP-Head Forward Tilt (*r* = 0.580), and HD-Torso Forward Tilt (*r* = 0.624) (all *p* < 0.05), and negatively correlated with IMP-Torso Side Tilt (*r* = −0.595, *p* = 0.041). Smash factor was positively correlated with HB-Trail Knee Flexion (*r* = 0.654), HD-Neck Rotation (*r* = 0.679), HD-Lead Knee Flexion (*r* = 0.799), IMP-Neck Rotation (*r* = 0.657), and IMP-Lead Knee Flexion (*r* = 0.667) (all *p* < 0.05); and negatively correlated with HD-Torso Rotation (*r* = −0.716), HD-Pelvis Rotation (*r* = −0.628), and IMP-Torso Rotation (*r* = −0.597) (all *p* < 0.05). Vertical Angle was negatively correlated with HB-Lead Knee Flexion (*r* = −0.600), TOP-Lead Knee Flexion (*r* = −0.755), and IMP-Head Forward Tilt (*r* = −0.582) (all *p* < 0.05). Axis was negatively correlated with HB-Neck Side Tilt (*r* = −0.628), TOP-Neck Side Tilt (*r* = −0.637), HD-Head Side Tilt (*r* = −0.611), HD-Lead Knee Flexion (*r* = −0.579), IMP-Head Side Tilt (*r* = −0.600), and IMP-Neck Rotation (*r* = −0.617) (all *p* < 0.05); and positively correlated with IMP-Torso Rotation (*r* = 0.668) and IMP-Pelvis Rotation (*r* = 0.642) (both *p* < 0.05). RMP was positively correlated with HD-Head Rotation (*r* = 0.595, *p* = 0.041) and negatively correlated with HD-Lead Knee Flexion (*r* = −0.603, *p* = 0.038).

## 4 Discussion

The present study investigated differences in joint angles at key swing moments during driver, 5-iron, and 7-iron in twelve female professional golfers, as well as their relationships with shot performance. The findings provide quantitative evidence to support swing technique training and performance enhancement. The results showed that shot performance differed significantly among clubs, consistent with the structural characteristics and functional roles of each club type, and in agreement with established knowledge of golf biomechanics and previous studies (27). The driver produced significantly greater carry, CHS, and smash factor than irons. Shaft length directly determines the swing arc radius; moreover, the driver clubhead is often made of titanium alloy, and the clubface compresses at IMP, creating a “trampoline effect” that converts more kinetic energy into ball speed rather than clubhead vibration (28). The 7-iron exhibited significantly greater RPM than the driver and 5-iron, and vertical angle followed a gradient of 7-iron > 5-iron > driver. The high vertical angle and RPM of the 7-iron provide better stopping ability when approaching the green (9, 29). No significant differences were found in axis among clubs. The mean spin axis was positive for the driver (1.10° ± 10.86°) and 5-iron (3.51° ± 6.96°), indicating a greater tendency for a rightward curve (fade), whereas the 7-iron showed a negative mean axis (−2.03° ± 4.67°), indicating a greater tendency for a leftward curve (draw). Shorter shafts were associated with smaller dispersion of axis values, suggesting easier shot shape control. This may be explained by the lower lofts of the driver (8°–12°) and 5-iron (23°–27°), where the vertical gear effect has a weaker corrective influence on axis, allowing side spin to be retained. In contrast, the larger loft of the 7-iron (31°–35°) generates more backspin through the gear effect at IMP, and its gyroscopic stability suppresses side spin (13).

The study found significant differences in head, torso, and knee angles among clubs, whereas lead elbow and wrist angles showed no significant differences across swing. This asymmetric variation pattern fully reveals that female professional golfers adopt a stable core movement strategy across different clubs, and only implement targeted and selective kinematic adaptations rather than overall posture reconstruction, which the primary adjustments occur in the trunk and lower limbs, while the upper extremities maintain a relatively stable control pattern. Differences in head angle were observed at HB and TOP. At both HB and TOP, the driver exhibited significantly smaller head forward tilt than the irons, and at TOP, driver head rotation was also significantly smaller. This difference likely reflects shaft-length effects, as the longer driver shaft may increase swing plane fluctuation and prompt golfers to reduce head forward tilt and rotation to stabilize gaze. Smaller head movement amplitude provides better visual field stability and body balance during the swing (30). Although this head stabilization strategy was observed across all clubs, its biomechanical relevance is greater in the driver because the longer shaft and larger swing arc amplify head displacement relative to the ball. The 7-iron similar head stability likely serves to maintain consistent fixation, although the smaller swing arc reduces perturbation to the visual-vestibular system. Overall, these patterns suggest that female professional golfers adaptively modulate head motion to balance distance generation with precision across clubs (31). At IMP, the driver showed significantly smaller torso forward tilt and torso side tilt but significantly greater torso rotation than the irons. The torso differences at impact likely reflect task specific mechanics, with the driver may emphasize rotation for speed and irons requiring more forward and side tilt for controlled contact. This reflects an adaptive adjustment in thorax movement: the driver requires greater thorax rotation to accumulate kinetic energy and transfer it to the club for increased CHS, whereas the irons, with their relatively smaller swing amplitude, increase torso forward tilt and side tilt which may be linked to more stable impact posture and potentially better shot precision. Additionally, at HB, driver torso rotation was smaller than that of the 7-iron, which may represent a deliberate energy conservation strategy during the backswing to avoid premature rotation and energy leakage. This finding aligns with previous studies identifying torso rotation as the core of the golf swing mechanics, suggesting that the female professional golfers' precise modulation of power transfer across different clubs (13). Regarding lead elbow and wrist angles, no significant differences, although some mean differences existed. Female professional golfers, through long-term systematic training, have developed a relatively fixed upper-limb swing control pattern. As distal segments of the swing, wrist and elbow angle adjustments may help maintain clubface stability (32) rather than to accommodate club differences. The consistent performance of elbow and wrist angles further verifies that elite female golfers retain unified distal swing habits, and do not change upper-limb control logic along with club switching. The relative stability of wrist and elbow angles may reflect a consistent distal control pattern across clubs and may be associated with greater swing repeatability in female professional golfers. Significant differences in knee angles were mainly concentrated on the lead knee, which may contribute to balance regulation and lower-limb posture control when female professional golfers adapt to different clubs. Collectively, only a subset of kinematic variables related to power generation and postural balance differed across clubs, whereas most joint angles remained stable, indicating a broadly consistent swing framework with selective adjustments in female professional golfers. At HB and TOP, the 5-iron showed significantly smaller lead knee rotation than the driver and 7-iron, indicating less internal rotation of the lead knee during the backswing. As a long iron, the 5-iron is technically more demanding than the 7-iron, balancing distance and accuracy. Golfers may subconsciously reduce lower-limb movement to increase stability. At HD, lead knee flexion was significantly smaller in the 5-iron than driver, suggesting that the lead knee is straighter at the start of the downswing. This insufficient knee flexion at HD indicates a delayed weight shift, which may compromise distance. Notably, at IMP, lead knee rotation was significantly greater in the 5-iron than driver, with the lead knee still in an internally rotated state, which may be associated with less efficient clubface delivery and a more rightward ball flight tendency.

Pearson correlation analysis revealed that the relationships between joint angles and shot performance were club-specific, identifying potential associations for technique training with different clubs in female professional golfers. Given the limited sample size and numerous statistical tests, all inter-group comparisons and Pearson correlation analyses are treated as exploratory and should be interpreted with caution. It is important to note that these correlations do not imply causation, as biomechanical, anthropometric, and equipment-related variables were not controlled for in this exploratory analysis. For the driver, TOP-lead forearm rotation was negatively correlated with carry and CHS, which merely indicates a statistical association, suggesting that excessive forearm rotation disrupts clubface stability. This is consistent with the modern professional approach of relying on body rotation to drive forearm rotation for clubhead speed release (33). At IMP, lead knee rotation was positively correlated with carry and smash factor and negatively correlated with RPM, indicating that greater lead knee external rotation at impact is associated with higher smash factor and longer carry. For the 5-iron, the swing appears to involve postural control of the torso and pelvis. IMP-torso forward tilt was negatively correlated with carry, and HB-head side tilt was negatively correlated with CHS, indicating that excessive torso forward tilt and head side tilt disrupt tend to be statistically associated with impaired body balance and potential shot deviations. At TOP, trail knee rotation (mean negative) was positively correlated with axis, suggesting that restricted external rotation of the trail knee may contribute to axis instability in the 5-iron. This finding aligns with McHardy et al., who reported that mid- and long-iron swings require greater centre of mass stability than driver, and any excess forward or side tilt can alter the angle of attack and compromise stability (34). For the 7-iron, the correlation results highlight potential relationships involving multi-joint coordination. At HD, lead knee flexion was positively correlated with smash factor and negatively correlated with axis and RPM. Maintaining a larger lead knee flexion angle is associated with higher smash factor and better launch axis orientation, although with lower RPM. This may be related to the preservation of spinal angle stability, which helps prevent premature extension that would reduce effective clubface loft. Appropriate RPM is essential for controlling stopping ability (23). At HB, pelvis rotation (mean negative) was positively correlated with carry, whereas at HD, torso rotation (mean negative) was negatively correlated with smash factor. This indicates that less pelvic rotation during the backswing and more torso rotation during the downswing are beneficial for 7-iron carry and smash factor. Elite golfers achieve a whipping effect through sequential braking of the pelvis, torso, and forearm before impact (7). If this interpretation is valid, training should therefore emphasise temporal separation between torso and pelvis rotation during the downswing rather than simply pursuing maximal rotational amplitude (35). However, given the correlational nature of this analysis, these interpretations should be viewed as hypothesis-generating rather than confirmatory. Future research employing multivariate or causal inference designs is warranted to substantiate these proposed mechanisms.

Several limitations of the present study should be acknowledged. First, the relatively small sample size (*n* = 12) may limit the generalizability of the findings; future studies should recruit larger samples and include golfers of varying skill levels to enable comparative analyses. Second, all participants used their own personal competition clubs rather than unified standardized experimental clubs, and inherent differences in club specifications may introduce unmeasured confounding variables that interfere with swing kinematic characteristics and hitting performance outcomes, which cannot be completely eliminated in this study. Third, the present study was restricted to joint angle measurements at four swing moments; future investigations should examine continuous kinematic and kinetic profiles across the full swing cycle to provide a more comprehensive characterization of movement patterns. Fourth, the absence of muscle activation data and ground reaction force measurements constrains our understanding of the underlying neuromuscular and force-application factors underlying these associations, and therefore limits mechanistic interpretation of kinetic chain function. Future research directions include: (1) integrating surface electromyography (sEMG) to examine muscle activation timing and coordination patterns across club types; (2) employing force platforms to quantify ground reaction forces and centre of mass displacement; (3) conducting longitudinal studies to assess the long-term effects of targeted technical interventions on swing kinematics; and (4) developing machine learning-based predictive models of shot performance to support individualized technical diagnosis and training program design. (5) Collecting more repeated trials in future 3D motion capture tests, and calculating intraclass correlation coefficient (ICC) and coefficient of variation (CV) to quantitatively evaluate measurement reliability and enhance data validity.

## 5 Conclusions

Carry, CHS, and smash factor were significantly greater for the driver than irons. RPM and vertical angle were significantly greater for the 7-iron than driver and 5-iron. No significant difference was observed in axis, yet shot control became easier as club length decreased. The driver exhibited smaller head forward tilt and rotation at HB and TOP than the irons to maintain visual stability and postural balance. At IMP, the driver was characterized by greater torso rotation but smaller torso forward and side tilt, whereas irons relied on increased torso forward and side tilt to achieve a descending strike. The 5-iron showed the smallest lead knee internal rotation at HB and TOP, insufficient lead knee flexion at HD, and persistent internal rotation at IMP, indicating technical deficiencies in regulation. Lead elbow and wrist angles remained relatively stable across clubs, reflecting consistent upper limb control patterns in female professional golfers. In the driver, excessive lead forearm rotation at TOP destabilized the clubface and reduced CHS and carry, while lead knee external rotation at IMP benefited smash factor. In the 5-iron, excessive torso forward tilt at IMP reduced carry, excessive head side tilt at HB lowered CHS, and excessive torso and pelvic rotation at HD decreased smash factor. In the 7-iron, maintaining greater lead knee flexion at HD enhanced smash factor and better launch axis orientation, while restricted pelvic rotation at HB helped increase carry. These findings suggest that club-specific training programs should be developed for female professional golfers, with targeted optimization of key joint angles including torso forward tilt, lead knee rotation, and lead forearm supination position.

## Data Availability

The raw data supporting the conclusions of this article will be made available by the authors, without undue reservation.
